# Cocaine

**Published:** 1893-11

**Authors:** Charles P. Briggs

**Affiliations:** Boston


### International Dental Journal.

Vol. XIV. November, 1893. No. 11.

### Original Communications.[1]

### COCAINE.[2]

BY CHARLES P. BRIGGS, M.D., D.M.D., BOSTON.

In my practice I find it necessary to use a great deal of cocaine; I use it principally for the removal of living pulps. For this purpose it is necessary to employ a twenty-per-cent. solution. Without much exaggeration, I may say that I have used quarts of a solution of this strength, and never, saving on two occasions, have I seen any unpleasant effects. On July 7, 1891, I met with a case which I considered one of cocaine-poisoning.

The patient was a young girl, thirteen years old. She had an exposed pulp in the left superior first molar which, as she was going to the mountains on the following day, it seemed desirable to remove with cocaine. As the tooth was so broken down that it was impossible to apply the rubber-dam, I enclosed the tooth as securely as I could in napkin and bibulous paper each time that I used the solution, and after each injection had the patient carefully rinse her mouth with water; I cautioned her, moreover, against swallowing. All went well, and I had nearly finished the removal of the pulp when the girl said to her mother, who was standing near us,

[1] The editor and publishers are not responsible for the views of authors of papers published in this department, nor for any claim to novelty, or otherwise, that may be made by them. No papers will be received for this department that have appeared in any other journal published in the country.

[2] Read before Harvard Odontological Society, July 1, 1893.

"Mother, I see grandmother standing there, and she tells me to be of good cheer." So strange a remark recalled the hallucinations often present in cocaine-poisoning. I looked at the patient, my attention up to that time having been given to the work in hand, and saw that her face was cyanotic. To my questions she said that she felt "queer," and complained of a strange sensation in her chest. I felt of her chest over the heart, and found the heart beating at a tremendous rate, so forcibly that it easily could be felt through the chest-wall. Now thoroughly alarmed, I placed her in a recumbent position, gave her inhalations of ammonia, subcutaneous injections of ether and brandy, and vigorously slapped her hands. I soon had the satisfaction of seeing her face regain a more natural color, and of finding that her heart-beats were coming down to a more regular and more normal rate. Several times the cocaine seemed to get the upper hand, and the heart would beat irregularly, rapidly, and violently, but extra stimulation would bring it back to a normal condition. It was, however, two hours before I considered it safe for her to rise from a reclining position. She felt dizzy and weak, and did not sleep any through the night following, her mother afterwards told me, but suffered no further inconvenience beyond a soreness of the arm at the points where I had injected the ether.

Since that time the removal of the pulp by cocaine has been an operation satisfactory as before in its results, but attended by a certain loss of nervous vitality on my part for which it is hard to find compensation. Not that the case could be considered gravely dangerous, but when I reflect that toxic effects have been observed after the application of three drops of a twenty-per-cent. solution to an exposed pulp, I am hindered in my work by a haunting fear that some day I may meet with symptoms not so easily controlled.

Once again I have had alarming symptoms; this time the rubber dam was in place. Inhalations of nitrite of amyl and internal administration of brandy obviated the symptoms, which it is fair to say might have been attributed to the apprehension of the patient and to the shock of the operation.

Basing my conclusions as to the dangers of cocaine upon my own experience, having seen but one case in a great many administrations where the effects plainly were to be ascribed to cocaine, I should say that, with reasonable precautions, it was safe to inject twenty-per-cent. solutions into the pulp-cavity; but when I read the literature on cocaine-poisoning, I question whether I may not have been unusually fortunate.

Dr. E. C. Kirk, in a valuable paper entitled "Local Anæsthetic

Nostrums," read before the First District Dental Society of New York, tells us that chemical analysis shows that most, if not all, of the thousand and one nostrums now in the market for the "painless extraction of teeth" contain cocaine; in nine, the analysis of which he submits, the amount runs from 1.46 (the smallest) to 5.68 per cent. To those who use these preparations it may be interesting to hear some of the testimony, for and against cocaine as a local anæsthetic, that I have been able to collect.

Opinions as to its effects are as much at variance as are the toxic symptoms. Where one investigator says that it is of inestimable benefit to mankind, another asserts that it is more pernicious in its effects than can be described. Among those who take an adverse view is Dr. Edmund Falk, of Berlin, who, in an article published in the *Therapeutische Monatshefte* of October, November, and December, 1891, gives a most exhaustive account of the effects of cocaine; in it he has given a list, in tabular form, of one hundred and seventy-six toxic cases, including ten deaths,—a collection made from all the available medical literature up to that time, including all the reported cases.

As the cases of poisoning from local application to mouth and throat and from subgingival injection are especially interesting to us, I give a translation of all the cases he has collected that come under these two heads. Dr. Falk's analysis of the one hundred and seventy-six cases is as follows:

When taken internally, the smallest dose after which toxic symptoms were observed was six- to nine-tenths of a grain. Death followed the injection of eighteen grains in one case and twenty-two grains in another, while very grave symptoms were seen after fifteen grains.

When applied locally, the poisonous dose depends upon the site of the application. Thus, while after injection into the bladder the smallest dose after which toxic symptoms were seen was fifteen grains, and an injection of eighty grains was not fatal, very alarming symptoms were observed after the injection into the urethra of fifteen drops of a three-per-cent. solution (one-third of a grain), and death resulted after the injection into the uterus of twelve grains, and after twenty-two grains into the rectum. After application to the nose of less than a quarter of a grain, after similar small doses to the external auditory canal, after eighteen-hundredths of a grain to the mucous membrane of the mouth, grave symptoms were seen. Painting of the larynx with a four-per-cent. solution caused poisoning. Extremely small doses to the conjunctival membrane, which seems to be peculiarly absorbent,—as, for example, six-hundredths of a grain,—caused poisoning. Death followed the subconjunctival injection of six-tenths of a grain.

Wölfler, in 1889, showed that in twenty-three cases of poisoning by subcutaneous injection nineteen were about the head, one in the larynx, and the three remaining were not to be considered, as the dose was excessive. This would tend to show that the head is an especially dangerous place for subcutaneous injection of cocaine. Hunt, in the *London Medical Recorder*, states that the mouth is the most dangerous place for its use. L. C. Anderson, in the *New York Medical Journal*, makes the same statement. But Falk's analysis of the poisonings from subcutaneous injections in his list does not confirm this. In twenty-eight of the cases the injection was made into the trunk, in nine into the extremities, and in seven into the head. Adding to these thirty-six cases of injection into the gums and five cases into the conjunctiva, we have forty-eight injections into the head and thirty-seven into other parts of the body. The amount injected into the extremities was not excessive,—that is, three grains was exceeded only five times, while about the head this amount, or more, was given six times. The smallest dose which produced toxic symptoms was two-thirds of a grain injected into the trunk, one-sixteenth of a grain into the forearm, and rather more than two-thirds of a grain into the head. From this it would seem that the site of the injection does not especially influence the production of toxic results.

The cases of poisoning from local application, however, show, as one would expect, that the dangerous dose depends upon the site of the application, owing, probably, to the varying rapidity with which the drug is absorbed in different parts of the body. The conjuctiva is a very rapid channel; after that the nose, mouth, and ear. In these situations one-third of a grain may generally safely be used. Then come the urethra, uterus, and rectum, for which the dose should not exceed one and a half to two grains. Fifteen grains may be injected into the bladder, if it is intact.

Besides depending upon the site of the application, whether local or subcutaneous, the poisonous dose seems to depend upon the ability of the individual to tolerate the drug. It must be said, however, that there are cases of poisoning in the list from doses which had previously been used in the same individual without untoward results.

Mannheim asserts that old, poorly-nourished individuals are peculiarly susceptible. As regards age, Falk's list shows that ten cases were between one and ten years of age, seventeen between ten and twenty, twenty-three between twenty and thirty, eighteen between thirty and forty, seven between forty and fifty, four between fifty and sixty, five between sixty and seventy, and three between seventy and eighty, the greatest number being between twenty and thirty. As to the type of patient, the list shows that nervous, anæmic individuals are peculiarly liable to evil after-effects. Lepin and Lewin say that this is so. L. C. Anderson, in the *New York Medical Journal*, says that patients of a cyanotic type and of sluggish respiration bear cocaine badly. Sleigh, in the *Buffalo Medical and Surgical Journal*, makes the same statement.

A summing up of Falk's conclusions would be that cocaine is unreliable and dangerous; that one can never feel sure that a dose which ordinarily may be considered safe may not be followed by alarming symptoms.

There are, however, as has been mentioned, those who hold a far different opinion of the matter. Among others is one whose name carries a great deal of weight,—Paul Reclus. Jules Auber, a disciple of his, in a pamphlet published in Paris in 1892, attempts to prove that cocaine, wisely administered, is as safe as any of the alkaloids. "There are to-day," he says, "in Paris hospitals hardly three services where cocaine is not constantly used, while in other parts of Europe, Lander, Wölfler, Albers, and others have come forward as its defenders." At a conference of German surgeons at Heidelberg, Albers expressed his astonishment that cocaine was not more widely used, and stated his conclusions as follows:

(1) Solutions of five per cent. are best for deep cutaneous incisions and for the removal of large tumors. Stronger solutions are unnecessary.

(2) Solutions of this strength, injected after Reclus's method, are preferable to chloroform, on account of their harmlessness and because of the greater rapidity of action.

Auber gives it as his opinion that the dread of cocaine arises from the fact that great errors have been made in the reports of cases; that symptoms have been grossly exaggerated; that many of the symptoms recorded may be ascribed to emotional causes. At this point it may be interesting to mention a case reported by Hugenschmidt. He was called to administer cocaine to a woman sixty-nine years of age, who was about to submit to a painful dental operation. He found her in a very excited and nervous condition, owing to the fact that her physician had told her that the operation (the injection of cocaine, that is) was a very dangerous one.

In this state of affairs, Hugenschmidt refused to give the injection; but persuaded by the woman to do so, he pretended to follow her request, but, as a matter of fact, injected ten drops of distilled water. In less than thirty seconds the patient complained of terrible pain in her head, rose, tottered to a sofa, and fell, saying, "I am dying!" She then fell into a state of syncope that lasted half an hour.

It is the attempt of Auber, in the pamphlet mentioned above, to prove that, with proper precautions, cocaine in hypodermic injections is not dangerous. At the writing of his paper, sixteen fatal cases had been reported. His contention is that none of these were due to a moderate dose of cocaine hypodermically administered. Two of the cases, he claims, are identical with two others in the list, reported as separate cases through mistake. A third case reported as fatal was in reality not fatal. Two other cases are thrown out, as death followed the ingestion, not the sub-cutaneous injection, of cocaine, and that in excessive doses. Of the eleven remaining cases, death was due in the first to the injection into the urethra of twelve grains; the injection was not submucous, and here again the dose was unwarrantable. In the second, death followed the injection of eight grains into the tunica vaginalis for hydrocele. The autopsy showed a serious valvular disease of the heart, to which, and to the excessive dose, the result could be ascribed. In the third case four injections of a five-per-cent. solution were made into the breast for mammary cancer, making a dose of three and a third grains. When most operations can be made with from eight- to nine-tenths of a grain, the dose may be considered excessive. The fourth case is the well-known one of Kolomin's, who was about to operate for ulceration of the rectum. Turning to one of his staff, he asked the dose of cocaine, and was told two to three grains. He injected twenty-four grains. Death followed in spite of all means for resuscitation. This death led to the suicide of Professor Kolomin.

The next three cases Auber skips lightly over, inasmuch as the cocaine was not given subcutaneously; but as they are all cases where the poison was absorbed through the mouth and throat, and as the solution used in two of them was of a strength usually considered safe, it may be interesting to hear of them somewhat in detail. The first of these three was that of a man whose larynx was sprayed three times with a four-per-cent. solution, the quantity unknown. Two hours afterwards he lost consciousness. His respiration was feeble, pulse twenty. Whiskey was injected under his skin, and in half an hour he regained consciousness. He gradually recovered, barring a feeling of weakness. Five days later two applications of a two-per-cent. solution were made. Two hours and a half afterwards the patient showed the same symptoms as before. Whiskey was injected, but he soon ceased to breathe. The heart continued to beat for some moments, but all efforts, including artificial respiration, failed to revive him.

The second case is reported by Dr. E. M. Thomas, of Leonardville, Kansas. He was called, October 23, 1885, to a woman thirty-nine years of age. He found her unconscious, with pulse thirty-five, irregular and intermittent, and respiration feeble. Temperature normal. Right pupil normal, left widely dilated. Right arm and the legs paralyzed. The left arm and upper part of the body twitched spasmodically. Twice during his stay she vomited parts of her meal of the evening before. Flow of saliva increased. In spite of all efforts, she died. He had applied a solution (four-per-cent., quantity unknown) to her gums for dental neuralgia.

The third case was that of a pharmacist, who, believing that he had diphtheria, applied powdered cocaine to his tonsils, at intervals, for seven hours. At the end of that time he manifested alarming symptoms and soon died.

The next case—the eighth—was that of a girl who was given a subcutaneous injection of four to twelve drops of a four-per-cent. solution (three-tenths of a grain). Forty seconds later she became unconscious, and died in one minute. She had a degeneration of the heart following scarlet fever, which, together with the effect of a dose of cocaine, ordinarily safe, added, proved fatal.

In the next case death followed a small dose subcutaneously administered; but the autopsy showed a cerebral hemorrhage which, to the satisfaction of the physicians present at the autopsy, was the cause of death.

The last case is one which will especially interest us, so an account of it is given in full. On August 7, 1890, Mlle. Delcambre went to the office of Dr. Bouchard, in Lille. Dr. Bouchard reports the case as follows:

" Mlle. X. came to my office August 7, 1890, to make an appointment for the following morning at seven o'clock. After making two injections of a one-per-cent. solution of cocaine, I proceeded to extract the two superior wisdom-teeth. The teeth were successfully extracted. After several minutes the patient expressed a desire to have me extract a third tooth on the left side of the lower jaw. I injected the rest of the cocaine in my syringe; after some minutes I extracted the tooth. The patient seemed quite comfortable, though a little weak. Not foreseeing any accident, I left her to get her a cup of coffee. On my return I found that she had fainted. I laid her on the floor. After loosening her dress and her corsets, I rubbed her, dashed cold water on her, and, in a word, did all that my therapeutics would suggest. Physicians called in haste tried the same means, and in addition gave the patient injections of ether, inhalations of nitrite of amyl and of oxygen. All was in vain. At the end of half an hour she died. At the autopsy a double cord was found tied so tight around her chest that it was impossible to get a scalpel between the skin and the cord. The medical experts and the judges with them have admitted that the patient, very much frightened, had had an attack of syncope, induced not by the cocaine, but by fear, which, thanks to the impediment offered to the respiration by the cord of discipline, had been fatal."

The *Journal für Zahnheilkunde*, reporting the case, states that the dose was one grain given in three doses.

Dr. Bouchard was acquitted of the charge of homicide, but fined a few francs for practising medicine without a license.

Summing up these cases, Auber asserts that in none of them was death due to the effects of cocaine when administered in a safe dose. There are unpleasant symptoms occasionally after cocaine, even in safe doses, but nothing that cannot easily be controlled.

The method of administration of the injections is of great importance. It is necessary to have no fear of the operation, for by a timorous demeanor the operator will suggest to the patient the possibility of danger. The patient should not be anæmic or hysterical, should have no heart-trouble. The clothing should be such as not to impede the respiration. It is of the utmost importance that the patient be placed in a horizontal position and kept there throughout the operation. Dujardin-Beaumetz has drawn attention to this point. He mentions several cases where the patients have suffered from vertigo, syncope, or sensory illusions when sitting up, but were free from these symptoms when lying down.

It is better to give the injection on a full stomach. It is necessary to have the patient drink a glass of whiskey half an hour before; and if during the operation—the operation for which the cocaine is used—he shows signs of pallor, he should be given another glass. The syringe should be aseptic. The cocaine should be dissolved at the time of the operation in water recently boiled. The dose should not exceed six, eight, or ten centigrammes (0.926, 1.235, and 1.543 grains respectively), which suffices for the most extensive operations. But the most important thing is the strength of the solution. It may be two per cent. for smaller operations which call for two to four centigrammes of cocaine; for those calling for six, eight, and ten centigrammes the solution should be one per cent.

For a long time Reclus has drawn the attention of surgeons to the fact that solutions of a low per cent. are, much out of proportion, less harmful than the more concentrated ones. At a meeting of the Société de Chirurgie, in December, 1891, he announced that he should make it a practice to use one-per-cent. solutions. In the first part of the year beginning January, 1892, he operated six hundred times without any alarming symptoms. Writing in the *Revue Scientifique*, March 26, 1892, he says,—

"The dangers of cocaine depend not only on the total amount of the alkaloid injected, but in a great measure upon the strength of the solution as well: the feebler the solution, the more the cocaine is diluted, the less are accidents to be feared. For example, ten centigrammes of cocaine in a one-per-cent. solution are much less to be feared than in a two-, and especially in a five- or ten-per-cent. solution. I have at this hour operated five hundred times, perhaps, when I have exceeded my maximum dose of ten centigrammes, using a one- or two-per-cent. solution.

"*Eh bien!* I should not have dared to inject the same amount in a ten-per-cent. solution.

"And this is easily understood. The solution injected under the skin is absorbed by the vessels which convey it to the nervous centres, where it exercises its harmful action. If the solution is a one-per-cent., it is ninety-nine parts water and one of cocaine that in a given time reaches the nervous centres; if it is a five- or ten-per-cent., it is five or ten parts cocaine that reach the brain in the same time. I insist on this point, for you must understand that in less than six years I have performed seventeen hundred and thirty-nine operations of all kinds, of all degrees of gravity, in all parts of the body, without having to deplore a single accident, while numbers of my colleagues—almost all, I might say—have been alarmed, if nothing more, by doses often more feeble than mine, but in stronger solutions."

Reclus himself, when using five-per-cent. solutions, had several accidents.

In injecting, it is necessary to observe the following rules laid down by Reclus. The injection should not be hypodermic, but endermic. The needle should be pushed along parallel with the surface; if there is resistance to the advance of the point, we may know that it is still in the derma. If the point slips easily along, we may infer that it has gone too deep, into the subdermal connective tissue.

M. Moty agrees with Reclus, that the dangers of cocaine depend upon the strength of the solution. He records the extraction of five to six thousand teeth, with cocaine, without accident.

Magitot (*British Medical and Surgical Journal*), following Reclus's teaching, concludes that cocaine is an excellent local anæsthetic, and should not be banished from surgery; that the dose should be proportionate to the extent of surface to be anæsthetized; that it should not be employed in patients suffering from chronic affections of the respiratory organs, or in those of well-marked neurotic diathesis; that patients should assume recumbent positions, and that it should be given in divided doses at intervals of some minutes.

Anton Bleichsteiner (*Wiener Klinische Wochenschrift*) mentions three thousand cases in two years, mostly for extraction of teeth, with no bad symptoms.

